# COmmuNity-engaged SimULation Training for Blood Pressure Control (CONSULT-BP): A study protocol

**DOI:** 10.1097/MD.0000000000023680

**Published:** 2021-02-05

**Authors:** Jennifer Tjia, Michele Pugnaire, Joanne Calista, Nancy Esparza, Olga Valdman, Maria Garcia, Majid Yazdani, Janet Hale, Jill Terrien, Ethan Eisdorfer, Valerie Zolezzi-Wyndham, Germán Chiriboga, Lynley Rappaport, Geraldine Puerto, Elizabeth Dykhouse, Stacy Potts, Andriana Foiles Sifuentes, Sylvia Stanhope, Jeroan Allison, Vennesa Duodo, Janice Sabin

**Affiliations:** a University of Massachusetts Medical School; b Center for Health Impact, Worcester; c Promoting Good, LLC, Upton, Massachusetts; d University of Washington, Seattle, Washington, USA.

**Keywords:** bias awareness, community engagement, implicit bias, medical education, simulation training

## Abstract

**Background:**

Healthcare professionals have negative implicit biases toward minority and poor patients. Few communication skills interventions target implicit bias as a factor contributing to disparities in health outcomes. We report the protocol from the COmmuNity-engaged SimULation Training for Blood Pressure Control (CONSULT-BP), a trial evaluating a novel educational and training intervention targeting graduate medical and nursing trainees that is designed to mitigate the effects of implicit bias in clinical encounters. The CONSULT-BP intervention combines knowledge acquisition, bias awareness, and practice of bias mitigating skills in simulation-based communication encounters with racially/ethnically diverse standardized patients. The trial evaluates the effect of this 3-part program on patient BP outcomes, self-reported patient medication adherence, patient-reported quality of provider communication, and trainee bias awareness.

**Methods:**

We are conducting a cluster randomized trial of the intervention among cohorts of internal medicine (IM), family medicine (FM), and nurse practitioner (NP) trainees at a single academic medical center. We are enrolling entire specialty cohorts of IM, FM, and NP trainees over a 3-year period, with each academic year constituting an intervention cycle. There are 3 cycles of implementation corresponding to 3 sequential academic years. Within each academic year, we randomize training times to 1 of 5 start dates using a stepped wedge design. The stepped wedge design compares outcomes within training clusters before and after the intervention, as well as across exposed and unexposed clusters. Primary outcome of blood pressure control is measured at the patient-level for patients clustered within trainees. Eligible patients for outcomes analysis are: English-speaking; non-White racial/ethnic minority; Medicaid recipient (regardless of race/ethnicity); hypertension; not have pregnancy, dementia, schizophrenia, bipolar illness, or other serious comorbidities that would interfere with hypertension self-control; not enrolled in hospice. Secondary outcomes include trainee bias awareness. A unique feature of this trial is the engagement of academic and community stakeholders to design, pilot test and implement a training program addressing healthcare.

**Discussion:**

Equipping clinicians with skills to mitigate implicit bias in clinical encounters is crucial to addressing persistent disparities in healthcare outcomes. Our novel, integrated approach may improve patient outcomes.

**Trial registration:**

NCT03375918

**Protocol version:**

1.0 (November 10, 2020)

## 1 Introduction

### 1.1 Rationale for the trial

Evidence suggests that healthcare professionals have negative explicit and implicit biases toward minority and poor patients,^[1,2]^ which can adversely affect clinical decision-making^[3–6]^ and interpersonal communication.^[7]^ Mitigating clinician bias through targeted training in bias awareness and interpersonal communication skills is a promising strategy for decreasing healthcare disparities for racially, ethnically, and socio-economically disadvantaged persons.^[8]^ Few rigorously designed trials of communication skills target implicit bias as a factor contributing to disparities in health outcomes.^[9,10]^

To address this gap, we designed a theoretically-grounded, multi-component, training intervention trial called “COmmuNity-engaged SimULation Training for Blood Pressure Control” (CONSULT-BP). The primary aim of the trial is to improve patient outcomes using a training program that combines knowledge acquisition, bias awareness development, and simulation-based communication practice with racially and ethnically diverse patients in order to better prepare clinicians for patient interactions in which implicit bias may affect healthcare outcomes. The clinical focus of the intervention is hypertension management because it represents a challenging public health priority,^[11,12]^ with healthcare disparities and clinical outcomes mediated, in part, by the quality of clinician-patient communication, bias^[13]^ and unsatisfactory patient experiences of care.^[14]^

The current educational arsenal to address racism and bias in healthcare has critical gaps, notably failing to incorporate personal bias awareness and evidence-based bias mitigating strategies into a program of practice and feedback from patients.^[10]^ We address these gaps in our trial by implementing a multi-component “knowledge, awareness, and practice” training model that involves community member and patient representation during the design and operationalization of the educational intervention. This protocol report provides a resource for others seeking to design a trial that evaluates and implements a training intervention to mitigate implicit bias in clinician-patient encounters.

### 1.2 Objectives

The aims of the clinical trial are to evaluate the effect of this 3-part, training program on:

1. patient blood pressure outcomes, reported in the electronic medical record (EMR);
2. self-reported patient medication and diet adherence, as measured by the Hypertension Medication Nonadherence Scale^[15]^ and the Blood Pressure Self-Care Scale;^[16]^
3. patient-reported quality of provider communication, as measured by the Health Care Climate Questionnaire;^[17]^
4. the trust sub-scale of the Primary Care Assessment Survey;^[18]^ and
5. trainee bias awareness, as measured by the Bias Awareness Scale.^[19]^

## 2 Intervention

### 2.1 Description of the CONSULT-BP educational intervention

#### 2.1.1 Core educational elements

The three (3) core elements of the educational and training intervention include:

1. trainee knowledge acquisition about healthcare disparities, implicit bias and racism;
2. trainee awareness of personal bias; and
3. provision of skills to mitigate bias, along with an opportunity to practice those skills with authentic standardized patient actors. (Table 1)

**Table 1:** COmmuNity-engaged SimULation Training for Blood Pressure Control (CONSULT-BP): Core Elements and Tailored Elements.

		Tailored Elements
Core Elements		Delivery
Key Domains	Key Content	CONSULT-BP
Knowledge	• Healthcare Disparities• Implicit Bias• Communication Skills to Mitigate Implicit Bias	*Online, self-administered*, learning modules
Self-Awareness	• Implicit Bias	4 IATs• Race-bias (2)• Race-compliance bias (2)
Skills Practice	• Hypertension management case with patient from vulnerable population	4 standardized patient encounters

A Standardized Patient (SP) is a person carefully recruited and trained to take on the characteristics of a real patient thereby affording learners an opportunity to practice and be evaluated on learned skills in a simulated clinical environment.

**Figure 1:**
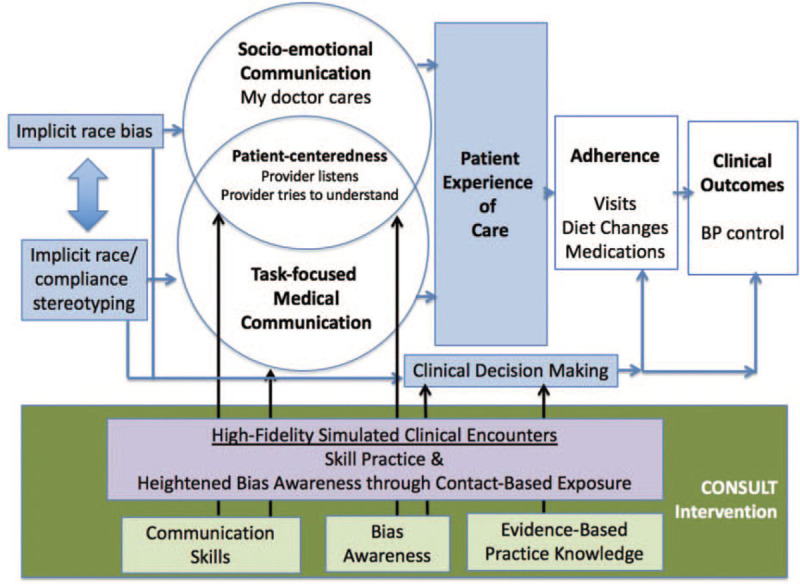
The CONSULT-BP intervention model.

To build knowledge, we used National Institute of Minority Health and Health Disparities-funded e-learning modules about health disparities, implicit bias, and patient-centered communication skills.^[20]^ We also deliver evidence-based practice knowledge about hypertension management, because the skills practice cases with SPs focus on hypertension management. To develop personal bias awareness, we use the Implicit Association Tests (IAT)^[21]^ with results feedback to the trainee. To develop skills, we present evidence-based strategies to mitigate bias previously tested in a randomized trial by Devine et al.^[22]^ Finally, trainees are provided an opportunity to practice those bias-mitigating skills using high-fidelity simulated clinical encounters with SPs.^[23]^ (Fig. 1)

#### 2.1.2 Theoretical framework

The educational intervention and its delivery were designed to reflect key features of an adaptation of Bennett's intercultural competency framework.^[24,25]^ In this theoretical model, to overcome *denial* about ones’ own implicit biases, learners need to first acquire knowledge and understanding about implicit bias. Then, to help learners develop *acceptance* of the effect of implicit bias on healthcare disparities, learners need to move toward *integration* and recognition of implicit bias within themselves and their clinical encounters. The ultimate goal is to motivate learners to acquire and apply effective communication skills in situations where implicit bias is likely to arise.

Applying the simulation-based training model of repeated practice and feedback for skills acquisition and progression to skill mastery,^[26]^ the CONSULT-BP training featured “mock” clinical encounters designed to “activate” trainee biases in the clinical care setting. To replicate the “authentic” experience of implicit bias in clinical care, the CONSULT-BP intervention developed face-to-face simulated clinical encounters with “acting” SPs recruited from local racial and ethnic communities as a foundational component of the program. These face-to-face interactions provided a “contact-based educational intervention.”^[25]^ Contact with groups for which one may hold biased attitudes may help reduce such bias.^[22,27]^

## 3 Methods and analysis

### 3.1 Trial design

This is a cluster randomized trial of the CONSULT-BP training using a stepped wedge design to evaluate the effectiveness of one-time CONSULT-BP training on patient outcomes. The stepped wedge design accommodates pre-existing training schedules to mitigate the effect of temporal trends in clinical skill proficiency. It also allows for all trainees to be assigned to the intervention, which was a pre-condition of involvement by the participating training programs, in order to meet training requirements. This design is statistically advantageous because all trainees have control and intervention periods, everyone serves as their own control, allowing for within and across participant comparisons based on data collection both before and after the intervention.^[28]^ Our model randomizes training times to one of five start dates within each academic year to accommodate pre-existing training schedules and to mitigate the effect of temporal trends in clinical skill proficiency.

### 3.2 Setting

This trial is being conducted at a single academic safety net hospital system that includes clinical delivery sites at the academic medical center, a community hospital, and three outpatient centers. Approximately 40% of medical center's primary care patients are low income and ∼27% non-white. The surrounding community includes 34% of residents that do not speak English at home, 21% are Hispanic/Latinx, and there are large newcomer immigrant and refugee populations. The medical center is a major regional training site for medical students, graduate nursing students, and postgraduate medical residents. The participating training programs include the internal medicine (IM), family medicine (FM) residency training programs, and the Doctor of Nursing Practice (DNP) nurse practitioner graduate school program. Participant recruitment started in September 2018 and we will enroll trainees and patients until August 2021. Data collection will continue until November 2021.

## 4 Participants

### 4.1 Eligibility criteria

#### 4.1.1 Trainee enrollment

Training programs assign their own trainees to participate in the intervention. Eligibility criteria for inclusion of trainee measures in the trial's outcomes analysis are:

1. Practice at a clinical site supported by the medical center's EMR to allow data collection for BP outcome measurement;

2. A 15-week clinical look-back period; and

3. No prior completion of the CONSULT-BP intervention. Trainees are provided a fact sheet and asked to opt-out of the study if they do not want their data used for outcomes analysis.

#### 4.1.2 Patient enrollment

Primary outcomes of blood pressure control are measured at the patient level for patients clustered within trainees as reported in the electronic medical record. Patients eligible for inclusion in the outcome analysis included being: 1. English-speaking; 2. Non-White racial/ethnic minority; 3. Medicaid recipient (regardless of race/ethnicity); 4. have hypertension. Exclusion criteria: We exclude patients with pregnancy, dementia, schizophrenia, bipolar illness, or other serious medical co-morbidity that would interfere with hypertension self-control, including enrollment in hospice. Secondary patient-reported outcomes of self-reported adherence and quality of communication with providers is measured from a subset of patients meeting the same eligibility criteria who are randomly recruited in periods before and after the training intervention.

### 4.2 Intervention assignment and recruitment

The educational intervention was integrated into the residency programs in IM and FM, and into the curriculum for DNP students. We are enrolling entire specialty cohorts of IM, FM, and NP trainees over a 3-year period, with each academic year constituting an intervention cycle. There are 3 cycles of implementation corresponding to 3 sequential academic years. Within each academic year, we randomize training times to 1 of 5 start dates using a stepped wedge design. Our biostatistician uses a random sequence generator to assign 5 groups of learners, from within each program, to 1 of 5 staggered intervention training dates within an academic year. Training program schedulers assign the trainees according to these assigned dates. The program is delivered over two in-person sessions, scheduled five weeks apart, to leverage a spaced learning design.^[29]^ The sessions were designed to minimize the time burden of the intervention on trainees outside of the classroom. As such, the in-person training sessions combined multiple educational components that trainees completed individually, but as part of an onsite group session. In each educational session, components included: “individual” online didactic modules, online IATs, face-to-face clinical practice simulation with SPs, and “group-based” facilitated debriefing sessions. Training in skills to mitigate the effects of implicit bias detailed by Devine et al^[30]^ was addressed as part of the group-based IAT debriefings that preceded case simulations with SPs. Each of the two, in-person sessions lasted about four hours. If trainee participants were unable to attend one of the sessions due to scheduling conflicts, we provided alternative dates for attendance.

### 4.3 Outcome measures and data collection

#### 4.3.1 Trainee measures

##### 4.3.1.1 Trainee demographics

We collect trainee age, gender, race/ethnicity, training year, whether they are US or foreign-born, and fluency in another language.

##### 4.3.1.2 Trainee IAT, explicit bias measures, bias awareness, and reaction to the IAT

Trainees are asked to complete online Race/Ethnicity IATs [Black/White, Latino/White]^[21]^ and Race/Ethnicity-Medical Compliance IATs [Black/White, Latino/White].^[31]^ Trainees were asked about their corresponding explicit beliefs and perceptions of what “other health professionals” believe about race/ethnicity and race/ethnicity-related medical compliance in order to assess trainees’ perception of their own bias as being “better than average".^[32]^ To assess trainees’ reaction to the IAT, three questions are included from Howell & Ratliff^[32]^ using a 4-point scale (Strongly disagree, Disagree, Agree, Strongly Agree) to measure the trainees’ degree of defensiveness to the IAT. Trainees also complete a 7-item Bias Awareness Scale, with items assessed on a 6-point scale (strongly agree to strongly disagree) and higher scores indicating greater bias awareness.^[19]^ These items were collected online during each of the in-person training sessions, including 2 IATs at each session.

##### 4.3.1.3 Assessment of Trainees by SPs

Community SPs complete standardized checklists of trainee performance measuring communication skills, perceptions of respect, emotional response, concern, empathy, listening skills, involvement in shared decision-making, engagement and partnership, BP measurement technique, and global performance.

#### 4.3.2 Patient Measures

The primary trial outcome is the change in BP as reported in the EMR. Clinical measurements are collected in the 15-week control period prior to CONSULT-BP training period and during a 20-week post intervention period. The *main outcome* measures in the primary analysis will be systolic and diastolic BP (mm Hg) from the EMR. Analyses will stratify BP control as defined by the Joint National Commission 8^[33]^: controlled HTN defined as SBP of <140/90 for most patients, or SBP < 150/90 for patients ≥60 years without a diagnosis of diabetes or chronic kidney disease, and uncontrolled hypertension as defined as greater than these values.

Secondary outcomes include patient self-reported adherence to visits, diet modification and antihypertensive medication use as measured by the BP Self-Care Scale,^[16]^ Hypertension Medication Nonadherence Scale,^[15]^ and quality of communication and trust measured in the Health Care Climate Questionnaire^[17]^ and the trust sub-scale of the Primary Care Assessment Survey. ^[18]^ These surveys are administered in clinic offices of participating trainees in the 15-weeks before and 20-week after the educational intervention. Following the stepped wedge design, all comparisons are before and after the intervention within patients nested within trainee and across trainees. Trained research assistants collect primary data from 5 patients per trainee in both the pre- and post-intervention periods. Patients are asked to rate the clinical interaction with the trainee on that day. Pictures of residents are included at the beginning of the questionnaire to confirm that the responses are being provided for the clinical trainee pictured.

### 4.4 Data management

We conducted data abstractions from the electronic medical record for primary outcomes assessment using a system that extracts data from the clinical database, reformats it and stores it on a separate analytics server to make querying the database more efficient and extract data without impacting clinical flow. Primary data collection from trainee self-report and patient questionnaires were entered into an electronic data capture system, RedCAP, using case report forms designed for this purpose, with the exception of the IATs which were directly collected by Project Implicit via an internet-based interface. Standardized patient assessments of trainee performance in the simulation lab are entered into the Learning Space data capture system used for all training and evaluation exercises at the clinical simulation lab.

### 4.5 Statistical analysis

For the primary outcome, we will compare SBP and DBP of patients to be seen by the participant trainees in the control period vs. the period after the training. For visits with more than one BP measurement, we will average the measurements within the visit. We will first compare the mean difference in SBP and DBP between the two study periods using mixed effects models (Model 1). We will then examine the linear trend of intervention effects using another set of mixed effect models (Model 2). Both models will adjust for patient and clinician characteristics, and include multi-level random effects to account for the correlation among patients in the same clusters of randomization, patients nested within clinicians and repeated measures from the same patients (same patients can be seen by the same clinician at control and after-training periods). Finally, we will conduct subgroup analysis by repeating the same analyses stratified by uncontrolled and controlled hypertension at baseline based on the definition in JNC 8.^[33]^

Secondary outcomes examine the role of trainee bias awareness and IAT on patient outcomes. We hypothesize that lower implicit bias and higher bias awareness are associated with lower post-intervention patient BP and better patient reported communication scores. The main outcomes for this analysis are patient reported outcomes (PROs) after the CONSULT-BP training. The main independent variables are trainees’ IATs (1. race and 2. race-compliance used in separate models) and bias awareness. The following mixed effects linear model (Model 3) will be used to examine the association between post- training PROs scores and clinicians’ IATs, adjusting for patient and clinician characteristics, and the correlation among patients nested in clinician (random effect). We will also control for the pre-training PRO score in the model. Since the pre-training score for each post-training patient is not available we will use the mean PRO score collected from the patients seen by clinician before training as a surrogate for the pre-training score. We will use separate models for race IAT and race-compliance IATs, stratified by Black-White race and Hispanic/Latinx-White race comparisons in separate models.

Descriptive analyses will characterize missing data frequency and associations with key trainee and patient characteristics. Variables associated with missingness will be included in analyses as covariates to account for effects from the missing pattern (missing at random).

### 4.6 Sample size calculation

For the primary outcome of BP change, we estimate that each resident will have at least 5 eligible patients per 5-week training block (assuming trainees see ∼25 patients per outpatient rotation block, of whom ∼40% have HTN (n∼10), and of whom ∼45% are low income). Thus, each trainee will see ∼10 eligible patients in the 3-block pre-intervention period, and ∼20 eligible patients in the 4 block post-intervention period. We estimate there will be approximately 205 enrolled trainees over a 3 year accrual period, yielding ∼205 × 10 pre-intervention observations (n = 2050) and 205×20 post-intervention observations (n = 4100) for all patients with HTN, and fewer (control∼1025 and intervention ∼2050) for participants with uncontrolled HTN (50%–70% in minority and poor populations).^[34]^ We will have >90% power to detect a 3 mmHg difference in patients at control and post-training periods.

### 4.7 Patient and public involvement — development of the intervention content and design

A unique feature of this trial was the engagement of academic and community stakeholders to design, pilot test and implement the educational and training program. To adapt the core educational elements and strategies into a feasible and acceptable intervention, we used a participation action research approach in which investigators collaboratively partnered with stakeholder participants. The goal was to work together to address system-specific issues affecting program operationalization.^[35]^*Community stakeholder partners* for educational design and delivery were racial/ethnic and socioeconomically diverse community leaders representing our local patient population. A local community health organization, with whom the study team had a long-standing research relationship, served as the community member recruitment liaison. Key *academic stakeholder partners* for educational design were School of Medicine and Graduate School of Nursing faculty from our target healthcare system. A separate community-based transformational change organization, with extensive experience in staff bias training and who worked closely with the medical center administrative leadership, was engaged to help design in-person facilitation around implicit bias. These partners worked together to design case simulation scenarios, a trainee performance evaluation checklist, and SP training protocols that were integrated into a cohesive, replicable training program. Finally, community members were recruited to be SPs, which created an opportunity for empowerment and equity, as community SP's provided direct skills feedback to healthcare trainees and contributed their “voice” as equal partners in the team effort to develop and refine the simulation scenarios. We sought to understand whether this creative and novel approach to communication skills training of healthcare professionals catalyzed the motivation of our learners to take their patient communication skills to a higher level of mastery through direct, objective, and specific feedback from individuals of color trained as SPs.^[25,36,37]^

### 4.8 Monitoring

The PI, in cooperation with the coinvestigators and the UMass Medical School Institutional Review Board, are monitoring the safety of the proposed project. We have created a data safety and monitoring plan and established formal monitoring procedures to closely monitor participant safety, data quality and study progress. A data monitoring committee is not needed since the study is minimal risk.

Monitoring for protocol adherence will be performed monthly to ensure early identification of poor performance. Specific parameters being monitored include psychological harm from the intervention, contacting patients who are ineligible for the study surveys; all adverse events are reported to the IRB. Our study protocol includes informed consent of the trainees for inclusion of their data in the outcome analysis, and informed consent for the patient questionnaires, and a Health Insurance Portability and Accountability Act (HIPPA) waiver for use of medical record data in the aggregated outcomes analysis.

## 5 Ethics and dissemination

### 5.1 Safety concerns

We obtained Institutional Review Board (IRB) approval at the UMass Medical School. To minimize research-associated risk and to protect the confidentiality of participant data, all investigators and staff involved in this project have completed extensive courses and passed certifying examination on the protection of human subjects in research through Collaborative Institutional Training Initiative training and HIPPA certification. The IRB conducts interim audits per their policies and procedures to assure compliance with standards of trial implementation.

### 5.2 Dissemination plan

Patient and organizational stakeholders will significantly contribute to the translation of the research findings into lay language. Additionally, our group has long-standing relationships with large, community-based organizations and will work closely with these stakeholders to develop and activate the infrastructure to disseminate our results through community-based outreach and academic venues. As per funder requirements, we will provide limited access to the final trial dataset via contractual agreements assuring data protections and security procedures. Furthermore, the protocol has been, and the results of this study will be, submitted to ClinicalTrials.gov and peer-review publications.

## 6 Discussion

In approaching the task of implicit bias education and training in healthcare, a gap exists between what is theoretically known to be effective^[24,25]^ and current practices. Appreciation of evidence that implicit bias exists in healthcare professionals has arguably been one of the most challenging aspects of addressing the persistent gaps in healthcare disparities. As modeled by Bennett and others,^[24,25]^ recognition and self-awareness of bias are the critical first steps in training to mitigate implicit bias in healthcare. Building on these priorities, the CONSULT-BP program specifically targets these first stages in the learning process of bias mitigation for healthcare professions.

Our effort to implement theoretically sound and novel approaches to clinician skill building for bias mitigation and improved patient-clinician communication across intercultural differences holds important implications for public health. While CONSULT-BP focuses on hypertension, the CONSULT-BP model is readily adaptable to other diseases, such as COVID-19, in which decisions for testing are emerging as being subject to bias.^[38]^ This adaptability of the CONSULT-BP model is as important as disparities in healthcare access and outcomes for racial, ethnic and poor populations is a persistent problem. Further, the Centers for Disease Control and Prevention recommends bias awareness and mitigation skills to address the public health crisis of COVID-19.^[39]^ To date, the domain of educational interventions for teaching about implicit bias in healthcare remains ripe for development. The CONSULT-BP protocol contributes useful insights into the design of bias education and training models for healthcare professionals, and provides a roadmap for others who share these goals and want to advance their own efforts.

## Acknowledgments

We would like to thank the community advisors, standardized patients, and trainees for their generous contributions to this project.

## Author contributions

**Conceptualization:** Jennifer Tjia, Michele Pugnaire, Jeroan Allison, Janice Sabin.

**Data curation:** Jennifer Tjia, Janice Sabin.

**Formal analysis:** Jennifer Tjia, Jeroan Allison, Janice Sabin.

**Funding acquisition:** Jennifer Tjia, Michele Pugnaire, Olga Valdman, Maria Garcia, Stacy Potts, Jeroan Allison, Janice Sabin.

**Investigation:** Jennifer Tjia, Joanne Calista, Olga Valdman, Maria Garcia, Majid Yazdani, Janet Hale, Jill Terrien, Ethan Eisdorfer, Valerie Zolezzi-Wyndham, Lynley Rappaport, Geraldine Puerto, Elizabeth Dykhouse, Sylvia Stanhope, Vanessa Duodo.

**Methodology:** Jennifer Tjia, Michele Pugnaire, Jeroan Allison, Janice Sabin.

**Project administration:** Jennifer Tjia, Joanne Calista, Nancy Esparza, Olga Valdman, Maria Garcia, Janet Hale, Germán Chiriboga, Lynley Rappaport, Geraldine Puerto, Stacy Potts, Andriana Foiles Sifuentes, Sylvia Stanhope, Janice Sabin.

**Resources:** Joanne Calista, Nancy Esparza, Olga Valdman, Maria Garcia, Janet Hale, Lynley Rappaport, Stacy Potts, Sylvia Stanhope, Jeroan Allison, Janice Sabin.

**Supervision:** Jennifer Tjia, Joanne Calista, Olga Valdman, Sylvia Stanhope.

**Writing – original draft:** Jennifer Tjia, Janice Sabin.

**Writing – review & editing:** Jennifer Tjia, Joanne Calista, Nancy Esparza, Olga Valdman, Maria Garcia, Majid Yazdani, Janet Hale, Jill Terrien, Ethan Eisdorfer, Valerie Zolezzi-Wyndham, Germán Chiriboga, Lynley Rappaport, Geraldine Puerto, Elizabeth Dykhouse, Stacy Potts, Andriana Foiles Sifuentes, Sylvia Stanhope, Jeroan Allison, Vennesa Duodo, Janice Sabin.
